# Investigation of Hepatitis E virus seroprevalence in fattening pigs in Taiwan

**DOI:** 10.3389/fvets.2026.1935236

**Published:** 2026-09-09

**Authors:** Chia-Chun Chang, Hui-Ping Hsiao, Min-Yuan Chia, Yen-Chen Chang, Shin-Wu Liu, Hsiu-Ya Yang, Ter-Hsin Chen, Chienjin Huang, Chia-Yu Chang

**Affiliations:** 1 Graduate Institute of Microbiology and Public Health, College of Veterinary Medicine, National Chung Hsing University, Taichung, Taiwan; 2 Department of Veterinary Medicine, College of Veterinary Medicine, National Chung Hsing University, Taichung, Taiwan; 3 Graduate Institute of Molecular and Comparative Pathobiology, School of Veterinary Medicine, National Taiwan University, Taipei, Taiwan; 4 Graduate Institute of Veterinary Pathobiology, College of Veterinary Medicine, National Chung Hsing University, Taichung, Taiwan

**Keywords:** fattening stages, Hepatitis E virus, serological investigation, swine, Taiwan

## Abstract

Hepatitis E virus (HEV) is an important zoonotic pathogen and a major cause of acute viral hepatitis worldwide. Domestic pigs are the primary reservoir of zoonotic HEV genotypes 3 and 4, making surveillance in pig populations important for understanding HEV epidemiology. However, updated data on HEV seroprevalence in Taiwanese pig populations remain limited. This cross-sectional study investigated the seroprevalence of HEV among fattening pigs in Taiwan. A total of 356 serum samples were collected from 32 commercial farms across five major pig-producing counties during 2024–2025 and tested for anti-HEV IgG by ELISA. Serum samples with sufficient residual volume (*n* = 274) were further tested for HEV RNA using RT-qPCR, and no HEV RNA-positive samples were detected. The overall individual-level seroprevalence was 57.6% (205/356, 95% CI: 52.4–62.7%), while 90.6% (29/32) of farms had at least one seropositive pig. Seroprevalence increased significantly with production stage, from 44.4% in early fattening pigs to 58.8% in middle and 66.5% in late fattening pigs. Compared with early fattening pigs, the odds of seropositivity were higher in middle fattening stages (OR = 2.89, 95% CI: 1.50–5.59) and late (OR = 5.24, 95% CI: 3.62–7.58). These findings demonstrate widespread HEV seropositivity among fattening pigs in Taiwan, suggesting that HEV infection commonly occurs before market age. The absence of detectable HEV RNA in serum is consistent with the transient nature of viremia reported in previous studies. Continued surveillance integrating both serological and molecular approaches, particularly including fecal samples for molecular testing, is warranted to better characterize HEV epidemiology in Taiwanese pig populations.

## Introduction

Hepatitis E virus (HEV) infection is one of the most important zoonotic and foodborne diseases, and is currently a major global cause of acute viral hepatitis in humans (1). According to the World Health Organization (WHO), HEV is responsible for approximately 20 million infections annually, resulting in thousands of deaths, and accounts for an estimated 5.4% of the disability-adjusted life years (DALYs) (1). Consistent with its zoonotic nature, serological surveillance has revealed that the host range of HEV is broad, including pigs, rodents, birds, dogs, cattle, deer, camels, sheep, and goats; however, clinical manifestations vary among host species (2). In humans, HEV infection can cause hepatic dysfunction, jaundice, vomiting, abortion or stillbirth in pregnant women, and chronic hepatitis or cirrhosis in immunosuppressed individuals (3–5). In contrast, in pigs and other mammalian species, HEV infection usually leads to mild or no clinical signs (6).

As a member of the genus *Orthohepevirus* within the family *Hepeviridae*, HEV harbors a positive-sense, 7.2-kb, single-stranded RNA genome that encodes three open reading frames (ORFs) (7). Based on the phylogenetic analysis, eight genotypes (HEV-1 to HEV-8) have been described (8). Among these, genotypes 1 to 4 are more prevalent, whereas genotypes 5 to 8 are relatively uncommon. Specifically, genotypes 1 (HEV-1) and 2 (HEV-2) are restricted to humans and are mainly transmitted via the fecal-oral route, causing large waterborne outbreaks in resource-limited and developing regions (8, 9). In contrast, genotypes 3 (HEV-3) and 4 (HEV-4) are zoonotic, infecting a wide range of mammals, with domestic pigs and wild boars serving as the primary reservoirs (10). Genotypes 5 (HEV-5) and 6 (HEV-6) have been incidentally identified in wild boars in Japan (11, 12), whereas genotype 7 (HEV-7) and 8 (HEV-8) have been detected in camels in the United Arab Emirates and China, respectively (13, 14). To date, no human infections with HEV-5, HEV-6, or HEV-8 have been reported; however, a chronic HEV-7 infection has been documented in an immunosuppressed individual, thereby highlighting its zoonotic potential (15).

In developed countries, zoonotic HEV-3 and HEV-4 are more frequently reported in humans than other HEV genotypes (9). Since domestic pigs are recognized as the principal reservoirs of HEV-3 and HEV-4 (16), most human infections are attributed to zoonotic transmission, primarily through the consumption of undercooked meat products or occupational exposure in pork-related industries (17–20). Within pig populations, transmission of HEV is primarily mediated through the fecal-oral route (21), involving ingestion of contaminated manure, water, fomites, or feed, as well as direct or indirect contact with infected animals. Following weaning, the decline of maternal antibodies and increased environmental exposure associated with mixing and housing changes contribute to heightened susceptibility (22). Consequently, factors such as inadequate biosecurity and concurrent infections that may compromise host immunity also facilitate sustained HEV circulation within herds.

Previous studies have demonstrated that HEV infection in pigs typically occurs between 8–15 weeks of age, with seroconversion characterized by an initial IgM response followed by IgG development (22–24). By the late fattening stage (>22 weeks of age), seroprevalence is usually high, ranging from approximately 40% to 70% across countries and regions (25, 26). Viral RNA is less frequently detected at slaughter age, however, reported positivity rates vary considerably among studies, geographic regions, and sample types (27–31). Although circulating viremia is generally transient and HEV RNA is infrequently detected in serum at slaughter age, viral RNA may still persist in the liver, bile, or other tissues and can also be shed in feces in a subset of pigs (32), posing particular food safety and occupational exposure concerns in Taiwan, where pork consumption is high. This age-dependent pattern identifies fattening pigs as the critical population for investigating HEV epidemiology, infection dynamics, and food safety risks.

In Asia, previous studies have consistently reported a high HEV seroprevalence in pig populations across different production stages (33–36). In Taiwan, an earlier study by Hsieh et al. reported a HEV seroprevalence of 37% in pigs, while a more recent investigation confirmed the presence of HEV RNA in slaughter-age pigs (30, 37). Collectively, these studies demonstrate that HEV infection has been widely documented in Taiwanese pig populations and underscore the need for updated epidemiological surveillance. To address this knowledge gap, this cross-sectional study was conducted to determine the seroprevalence of anti-HEV antibodies among fattening pigs from commercial farms in Taiwan. In addition, serum samples with sufficient residual volume were examined for HEV RNA by RT-qPCR to investigate the presence of circulating viremia at the time of sampling. This study provides updated baseline data to improve the understanding of HEV epidemiology in Taiwanese commercial pig populations, thereby supporting future surveillance and risk assessment.

## Material and methods

### Study design and sample collection

A cross-sectional study was conducted to determine the seroprevalence of anti-HEV antibodies among fattening pigs in Taiwan. Serum samples were obtained through diagnostic-based sampling from pigs submitted to two veterinary diagnostic services [Animal Disease Diagnostic Center (ADDC) of National Chung Hsing University and National Taiwan University] between 2024 and 2025. A total of 32 commercial pig farms across five major pig-producing counties were included (Table 1): Changhua (*n* = 6), Yunlin (*n* = 11), Chiayi (*n* = 2), Tainan (*n* = 5), and Pingtung (*n* = 8). These counties represent major pig-producing counties in Taiwan, where a substantial proportion of the country's commercial pig farming is concentrated. Pigs were classified into three production stages: early fattening (15–17 weeks), middle fattening (18–21 weeks), and late fattening (22–25 weeks). Farms were further categorized according to total pig inventory into three operational herd-size groups: small (< 3,000 pigs), medium (3,000–10,000 pigs), or large (>10,000 pigs). Among the 32 farms, 15 implemented sampling across two fattening stages, whereas the remaining 17 farms were sampled at a single fattening stage. At each sampling event, approximately 5–10 pigs were sampled per stage, yielding a total of 356 serum samples. Blood samples were collected via jugular venipuncture by licensed veterinarians, centrifuged at 3,000 rpm for 20 min to separate serum, and stored at −20 °C until analysis.

**Table 1:** Characteristics of pig farms and serum samples included in this study.

Variable	Category	No. of farms	No. of serum samples
County	Changhua	6	81
	Yunlin	11	123
	Chiayi	2	28
	Tainan	5	40
	Pingtung	8	84
Farm size	Small (< 3,000 pigs)	10	84
	Medium (3,000–10,000 pigs)	9	104
	Large (>10,000 pigs)	7	85
	Unavailable	6	83
Fattening stage	Early (15–17 weeks)	18^#^	126
	Middle (18–21 weeks)	8^#^	51
	Late (22–25 weeks)	24^#^	179

^#^Fifteen farms contributed samples from more than one fattening stage, including 13 farms sampled during both the early and late fattening stages and 2 farms sampled during both the middle and late fattening stages.

### Evaluation of serological anti-HEV antibody by ELISA

To determine the seroprevalence of anti-HEV IgG antibodies in pigs, serum samples were tested using the ID Screen^®^ Hepatitis E Indirect Multi-species ELISA kit (Innovative Diagnostics, Grabels, France). The assay employed paired antigen-coated (test) and non-coated (control) wells for each sample, with the antigen-coated wells containing recombinant HEV genotype 3 capsid protein. Positive and negative control sera supplied by the manufacturer were included in each assay. Following the manufacturer's instructions, serum samples were diluted 1:20 with the provided dilution buffer and incubated in the microplates for 45 min at room temperature with regular shaking. After washing, a multi-species horseradish peroxidase-conjugated secondary antibody was added and incubated for 30 min at room temperature. Following a second wash step, tetramethylbenzidine (TMB) substrate was added and incubated for 15 min before the reaction was terminated with stop solution. Optical density (OD) was measured at 450 nm using an ELISA reader (MRX TC II Microplate Absorbance Reader, Dynex Technologies, VA, USA). The sample-to-positive (S/P) ratio was calculated as follows:


$$\begin{array}{l} \frac{Sample\;OD_{Test\;well}-{Sample\;OD}_{Control\;well}}{{Positive\;Control\;OD}_{Test\;well}-{Positive\;Control\;OD}_{Control\;well}}\times 100 \end{array}$$

According to the manufacturer's recommendations, samples with an S/P ratio ≥ 70 were considered positive, those with an S/P ratio between 60 and 70 were classified as doubtful, and those with an S/P ratio < 60 were considered negative.

### HEV RNA detection by RT-qPCR

Following serological testing, serum samples with sufficient residual volume (*n* = 274) were subjected to HEV RNA detection (Supplementary Table 1). Total nucleic acids were extracted from 50 μL of serum using the Optipure Viral Auto Kit (TANBead, Taoyuan, Taiwan) on the Maelstrom 4,800 Automated DNA/RNA Purification Platform (TANBead), according to the manufacturer's instructions. Extracted RNA was reverse transcribed into complementary DNA (cDNA) using the QuantiTect Reverse Transcription Kit (Qiagen, Hilden, Germany). HEV was then detected by SYBR Green-based real-time PCR using the QuantiNova SYBR Green PCR Kit (Qiagen, Hilden, Germany). The primers used were 5 -GGTGGTTTCTGGGGTGAC-3 (forward) and 5′-AGGGGTTGGTTGGATGAA-3 (reverse), which amplify a 69-bp fragment within the highly conserved ORF3 region of the HEV genome (38–40). Each 20-μL reaction mixture contained 10 μL of 2 × QuantiNova SYBR Green PCR Master Mix, 1 μL each of forward and reverse primers (10 μM), 2 μL of cDNA template, and 6 μL of PCR-grade water. Amplification was performed with an initial denaturation at 95 °C for 2 min, followed by 40 cycles of denaturation at 95 °C for 10 s and annealing/extension at 60 °C for 30 s. A melting curve analysis was performed at the end of amplification. A synthetic 67-bp HEV genotype 4 DNA fragment was included as the positive amplification control, and PCR-grade water was included as a no-template negative control in each run. Based on the current standard curve (Supplementary Figure 1), the practical detection limit was estimated to be approximately 10^2^ copies/reaction and the melting temperature is 81 °C.

### Statistical analysis

At the individual level, the seroprevalence of HEV antibodies among pigs was calculated as the proportion of anti-HEV IgG-positive pigs among all tested pigs, with corresponding 95% confidence intervals (CIs). Farm-level prevalence was calculated as the proportion of farms with at least one seropositive pig among all sampled farms, and 95% CIs were also estimated. To assess the association between production stage and anti-HEV IgG seropositivity, a mixed-effects logistic regression model was fitted with serological status (positive/negative) as the binary outcome, production stage as a categorical fixed effect, and farm as a random effect to account for clustering of pigs within farms. Early fattening pigs were used as the reference category, and odds ratios (ORs) with 95% CIs were reported. Model convergence was assessed based on the optimizer convergence status, and numerical stability was evaluated by comparing estimates obtained using 40 and 80 quadrature points. The farm-level intraclass correlation coefficient (ICC) was estimated from the variance of the farm-level random intercept using the latent-variable method. For farms sampled at two fattening stages, within-farm changes in seropositivity across stages were summarized descriptively. As a sensitivity analysis, the mixed-effects logistic regression was repeated after restricting the dataset to farms from which pigs were sampled at more than one production stage, using the same model specification as in the primary analysis. In addition, farms were grouped by total pig inventory into small (< 3,000 pigs), medium (3,000–10,000 pigs), and large (>10,000 pigs), and farm-level seropositivity was summarized using medians and interquartile ranges (IQRs) and visualized with plots. Among farms with available total pig inventory information, differences in farm-level seropositivity among the three total pig inventory categories were assessed using the Kruskal–Wallis test.

## Results

### Overall seroprevalence of anti-HEV IgG antibodies

A total of 356 serum samples were collected from fattening pigs in 32 commercial farms across five counties located in central and southern Taiwan. The overall seroprevalence of HEV IgG antibodies among the pigs was 57.6% (205/356, 95% CI: 52.4–62.7%). At the farm level, 29 of 32 farms tested positive for anti-HEV IgG, corresponding to a prevalence of 90.6% (95% CI: 75.8%−96.8%).

### Seroprevalence by geographic region

Farm-level and sample-level seroprevalence by county were summarized in Table 2. Anti-HEV IgG-seropositive pigs were identified on farms in all surveyed counties, with farm-level seroprevalence ranging from 80.0% to 100%. These findings demonstrated widespread HEV seropositivity among commercial pig farms in the surveyed regions. For descriptive purposes, sample-level seroprevalence was also calculated. At the individual level, seroprevalence ranged from 54.8% to 64.3% across counties, indicating a broadly similar distribution of anti-HEV IgG seropositivity among the major pig-producing regions of Taiwan.

**Table 2:** HEV seroprevalence in pigs by county in Taiwan.

County	No. of farm sampled	No. of positive farm	Farm-level prevalence %	No. of total sample	No. of positive sample	Sample-level prevalence %(95% CI)
Changhua	6	5	83.3	81	46	56.8 (45.9–67.0)
Yunlin	11	10	90.9	123	73	59.3 (50.5–67.6)
Chiayi	2	2	100.0	28	18	64.3 (45.8–79.3)
Tainan	5	4	80.0	40	22	55.0 (39.8–69.3)
Pingtung	8	8	100.0	84	46	54.8 (44.1–65.0)
**Summary**	**32**	**29**	**90.6**	**356**	**205**	**57.6 (52.4–62.7)**

### Association of production stage with anti-HEV IgG seropositivity

Anti-HEV IgG seroprevalence increased progressively with production stage, rising from 44.4% (56/126) in early fattening pigs to 58.8% (30/51) in middle fattening pigs and 66.5% (119/179) in late fattening pigs (Table 3). To assess the association between fattening stages and anti-HEV IgG seropositivity, a mixed-effects logistic regression model was fitted with fattening stage included as a fixed effect and farm included as a random effect to account for clustering of pigs within farms. Compared with early fattening pigs, pigs in the middle fattening stage had higher odds of anti-HEV IgG seropositivity (OR = 2.89, 95% CI: 1.50–5.59, *P* = 0.0016), whereas pigs in the late fattening stage had the highest odds of seropositivity (OR = 5.24, 95% CI: 3.62–7.58, *P* < 0.001). The model converged successfully without convergence warnings. Increasing the number of quadrature points from 40 to 80 resulted in changes of less than 0.3% in the estimated ORs, indicating numerical stability of the model estimates. The farm-level ICC was 0.463. A sensitivity analysis restricted to the 15 farms sampled at more than one production stage showed results consistent with the primary analysis, with higher odds of seropositivity in middle (OR = 4.62, 95% CI: 1.33–16.05) and late fattening pigs (OR = 7.28, 95% CI: 3.23–16.41) compared with early fattening pigs. Overall, anti-HEV IgG seroprevalence increased significantly with advancing production stage.

**Table 3:** Seroprevalence of HEV in pigs by production stage.

Production stage	Seropositive/total	Prevalence % (95% CI)	Odds ratio (95% CI)	*P*-value
Early	56/126	44.4 (36.1–53.2)	1	-
Middle	30/51	58.8 (45.2–71.2)	2.89 (1.50–5.59)	0.0016
Late	119/179	66.5 (59.3–73.0)	5.24 (3.62–7.58)	< 0.001

### Within-farm changes in seropositivity across fattening stages

**Figure 1:**
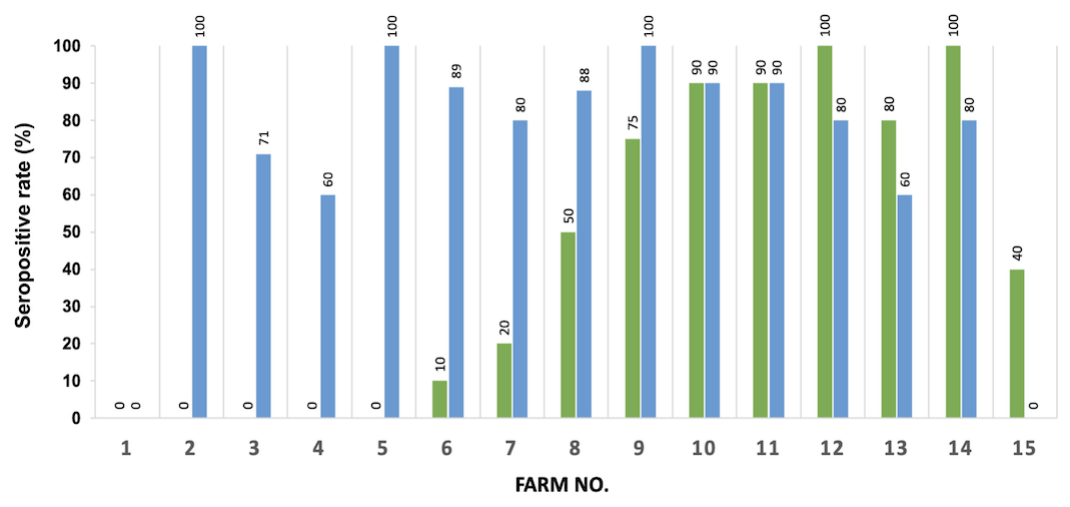
Changes in anti-HEV IgG seropositivity across fattening stages within individual farms. Fifteen farms (no. 1–15) were sampled at two fattening stages, including the late fattening stage and either the early or middle fattening stage. Green bars represented the seropositivity among pigs sampled at the early or middle fattening stage, whereas blue bars represented those sampled at the late fattening stage.

Among the 32 farms, 15 (farms no. 1–15) were sampled at two production stages, allowing within-farm comparisons of anti-HEV IgG seropositivity. All 15 farms were sampled during the late fattening stage, with an additional sampling performed during either the early or middle fattening stage. To avoid comparisons between adjacent weeks, samples from the middle and late fattening stages were collected with a minimum interval of four weeks. As shown in Figure 1, only one farm (farm no. 1) remained completely seronegative across both sampling stages. Eight farms showed increased seropositivity at the late fattening stage, four showed decreased seropositivity, and two maintained consistently high seropositivity (90%) across both sampling stages. Among the eight farms with increasing seropositivity, four (farms no.2, no.3, no.4 and no.5) demonstrated seroconversion from negative to positive status. These findings demonstrated considerable variation in seropositivity patterns among farms across production stages.

### Seroprevalence according to farm size

**Figure 2:**
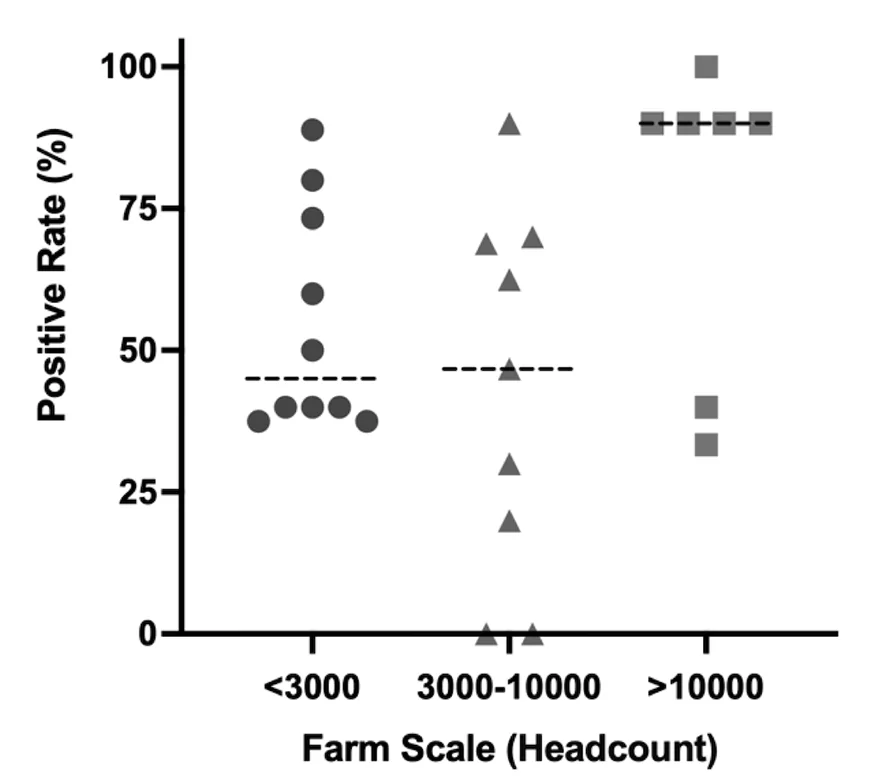
Farm-level anti-HEV IgG seropositivity according to farm size. Each point represented the seropositivity of an individual farm. Farms were categorized into three groups based on total pig inventory: small (< 3,000 pigs; *n* = 10), medium (3,000–10,000 pigs; *n* = 9), and large (>10,000 pigs; *n* = 7). Dashed horizontal lines represent the median seropositivity within each farm size category.

The farms were categorized into three groups based on total pig inventory: small (< 3,000 pigs), medium (3,000–10,000 pigs), and large (>10,000 pigs). Total pig inventory information was available for 26 of the 32 farms, including 10 small, 9 medium, and 7 large farms. Sample-level anti-HEV IgG seropositivity according to farm size is shown in Figure 2. Small farms had a median seropositivity of 45.0% (IQR: 40.0%−73.3%), with 6 of 10 farms exhibiting seropositivity ≤ 50%. Medium farms had a median seropositivity of 46.7% (IQR: 10.0%−69.4%), with 5 of 9 farms exhibiting seropositivity ≤ 50%. Large farms had a higher median seropositivity of 90.0% (IQR: 40.0%-90.0%), whereas only 2 of 7 farms exhibited seropositivity ≤ 50%. A higher median seropositivity was observed among large farms, although substantial variability remained within each farm size category. However, according to Kruskal–Wallis test, farm-level seropositivity did not differ significantly among the three total pig inventory categories [H(2) = 5.28, *P* = 0.071].

### Detection of HEV RNA in serum samples

Following serological testing, 274 of the 356 serum samples had sufficient residual volume for molecular analysis by RT-qPCR (Supplementary Table 1). HEV RNA was not detected in any of the tested serum samples. The remaining 82 serum samples were excluded from molecular analysis because of insufficient residual sample volume.

## Discussion

Hepatitis E virus (HEV) is an important zoonotic pathogen, with domestic pigs serving as the principal reservoir of zoonotic HEV genotypes 3 and 4. Recent regional reports from Asia have indicated that the seroprevalence of HEV in swine populations could be higher than previously reported, with some countries showing notable increases, suggesting possible changes in viral epidemiology and transmission patterns (41). To obtain updated information from Taiwan, a total of 356 convenience serum samples from fattening pigs across 32 farms were collected for serological investigation and serum samples with sufficient residual volume were subsequently analyzed for HEV RNA by RT-qPCR. The overall anti-HEV IgG seroprevalence among fattening pigs in Taiwan was 57.6%, with seropositive pigs detected on 90.6% of the surveyed farms. Seropositivity increased significantly with advancing fattening stage, while descriptively higher median seropositivity was observed among large farms despite substantial variability among farms. However, HEV RNA was not detected in any of the 274 serum samples.

The only serological survey on HEV seroprevalence in Taiwan was published in 1999, in which 275 serum samples from pigs older than three months across ten farms were analyzed, yielding a seropositivity rate of 37% (37). Notably, that study employed a homemade ELISA based on the capsid protein (ORF2) derived from a human HEV strain (Sar-55) as the coating antigen. Although previous studies had demonstrated substantial antigenic cross-reactivity among different HEV genotypes (42), the use of a heterologous antigen might still influence the sensitivity and specificity of serological assays. The seropositivity observed in the present study (57.6%) was higher than the 37% reported previously. Nevertheless, differences in assay methodology, sampled pig populations, and potentially geographic coverage between the two studies may limit the reliability of direct comparisons of temporal changes in HEV seroprevalence in Taiwan. In the current study, a commercial ELISA kit (IDvet) based on genotype 3 HEV capsid protein was used, which was more closely related to swine HEV strains. The use of a homologous or genotype-relevant antigen may enhance antibody detection and contribute to the higher seropositivity observed.

Nevertheless, an actual increase in HEV seroprevalence over time could not be excluded. A similar temporal trend had been reported in Singapore, where a 10-year surveillance study of imported pigs demonstrated an increase in seroprevalence from approximately 35% during 2000–2017 to around 60% in 2018–2019 (41). Likewise, studies conducted across Asia had consistently reported moderate to high HEV seroprevalence in swine populations. Earlier investigations documented seroprevalence ranging from 52.0% to 66.4% in China, the Philippines, and Laos (34, 43–45). More recent reports after 2020 had reported comparable seroprevalence in Vietnam (58.5%) (46), China (approximately 58%) (33, 47), and northeastern India (51.0%) (36), although lower prevalence had been observed in Indonesia (23.5%) and Mongolia (35.5%) (48). Taken together, these findings indicated that the seroprevalence observed in the present study is comparable to that reported in many Asian countries, although direct comparisons should be interpreted cautiously because of differences in study design, sampling strategy, pig age, and serological assays.

To investigate potential factors influencing HEV seropositivity, samples were categorized into early, middle, and late fattening stages, and information on the farm rearing scale/size was collected. To account for the unbalanced sampling across farms and production stages, a mixed-effects logistic regression model was applied to evaluate differences in seropositivity among fattening stages. The odds of anti-HEV IgG seropositivity increased significantly with advancing fattening stage, with pigs in the middle and late fattening stages having 2.89- and 5.24-fold higher odds of seropositivity, respectively, than pigs in the early fattening stage. The higher seropositivity observed in late fattening pigs likely reflects cumulative exposure to HEV over the production period. As pigs progress through production stages, they have a longer opportunity to become infected, and anti-HEV IgG generated following infection may persist after the transient viremic phase, resulting in a progressively greater proportion of seropositive animals at later production stages. An age-related increase in HEV seroprevalence had been consistently reported in pig populations. Previous studies in other geographical regions had shown that seropositivity rose progressively with production stage, increasing from 37.6% in growing pigs to 64.1% in finishing pigs and reaching 78.8% in breeding pigs (34); Similar patterns have also been observed in other studies, where seroprevalence increased from 41.01% in grower pigs to 62.41% in sows (33). Longitudinal investigations further indicated that most pigs become infected between 8 and 15 weeks of age, which coincided with the waning of maternally derived antibodies (24, 25, 49).

This study also provided an exploratory assessment of whether total pig inventory might be associated with farm-level anti-HEV IgG seropositivity. Although large farms showed a descriptively higher median seropositivity than small and medium-sized farms, the difference was not statistically significant. Moreover, this analysis included only 26 farms, comprising 10 small, 9 medium, and 7 large farms, which limited the statistical power. The wide and substantially overlapping IQRs further indicated considerable within-category variability. Nevertheless, the observed tendency toward higher seropositivity in larger farms may warrant further investigation. Several factors associated with larger production systems could potentially contribute to increased opportunities for HEV transmission, including higher stocking densities, more frequent introduction and movement of pigs, greater mixing of animals from different groups or sources, more frequent movement of personnel and equipment between production units, and greater logistical complexity in maintaining consistent hygiene and biosecurity measures across the farm. In addition, differences in biosecurity practices, production management, housing systems, and herd replacement or pig-flow strategies may contribute to variation in seropositivity between individual farms, independent of herd size itself. Taken together, the pattern observed here may represent a potential trend that should be further evaluated in larger studies with sufficient numbers of farms across total pig inventory categories.

In addition to serological testing, HEV RNA was examined in 274 serum samples with sufficient residual volume; however, no detectable viremia was identified. This finding is consistent with previous studies demonstrating that serum viremia is typically transient, with an estimated mean duration of approximately 10.5 days, and is rarely (< 3%) detected in pigs approaching slaughter age (22, 50). In contrast, fecal viral shedding may persist for a longer period (~7–50 days) after viremia has resolved (22). Accordingly, serum is likely a suboptimal specimen for molecular detection of HEV in pigs at slaughter age, as the relatively short viremic phase may have already resolved while fecal shedding persists. Reliance on serum alone may therefore underestimate the presence of ongoing or recent HEV infection. Consistent with this infection pattern, Liao et al. (30) reported that approximately 25% of slaughter-age pigs in Taiwan remained positive for HEV RNA in feces, despite the low likelihood of detecting circulating virus in serum (30). Therefore, the lack of detectable HEV RNA in serum in the present study does not necessarily indicate the absence of HEV circulation within the surveyed pig population, but may instead reflect the limited temporal window for detecting viremia at slaughter age. These findings further supported the complementary roles of serological and molecular approaches in characterizing HEV epidemiology in commercial pig herds.

While the present study provided insights into HEV seroprevalence in Taiwanese pigs, several limitations should be acknowledged. First, the samples were obtained through diagnostic-based sampling and were unevenly distributed among farms and production stages, which may have introduced sampling bias. Pigs submitted for diagnostic testing may have had underlying health conditions that could potentially influence their immune status and susceptibility to infection. Moreover, because the samples were derived from diagnostic submissions rather than a population-based random sampling framework, the sampled farms and pigs may not fully represent the general pig population in Taiwan. Therefore, the observed seroprevalence and the high farm-level seropositivity should be interpreted in the context of the diagnostic-based sampling strategy used in this study. Second, detailed information on housing systems, farm management practices, biosecurity measures, and equipment and animal movement was unavailable, precluding further evaluation of potential farm-level risk factors associated with HEV seropositivity. Third, the relatively small number of middle fattening pigs may have reduced the precision of the corresponding OR estimate, as reflected by its relatively wide 95% confidence interval. Finally, because this was a cross-sectional study, the temporal dynamics of HEV infection could not be determined. Nevertheless, the consistent age-related increase in anti-HEV IgG seropositivity observed across production stages provided updated epidemiological evidence of widespread HEV infection in commercial pig populations in Taiwan.

In conclusion, this study provided updated epidemiological data on HEV infection in Taiwanese commercial pig populations. Anti-HEV IgG seroprevalence was substantially higher than that reported previously and increased significantly with advancing fattening stage. Although HEV RNA was not detected in serum samples, this finding was consistent with the transient nature of viremia in pigs. The observed variation in seropositivity among farms suggested that farm-level factors might influence HEV epidemiology and warranted further investigation. Future surveillance integrating serological and molecular data with detailed information on farm management practices, biosecurity measures, cleaning and disinfection protocols, and pig flow management, will provide a more comprehensive understanding of HEV epidemiology in commercial pig populations in Taiwan.

## Data Availability

The raw data supporting the conclusions of this article will be made available by the authors, without undue reservation.
