# Supplementary material for: Investigation of Hepatitis E virus seroprevalence in fattening pigs in Taiwan
Source: Front Vet Sci. 2026 Sep 9;13:1935236. doi: 10.3389/fvets.2026.1935236 (PMC13598322; doi:10.3389/fvets.2026.1935236)
Supplement: Supplementary file 1 [file Table_1.DOCX]

**Supplementary Table 1. Distribution of serum samples included and excluded from HEV RNA testing according to production stage and anti-HEV antibody status**

| Production stage | Sample included | | | | | Sample excluded | | | | |
| --- | --- | --- | --- | --- | --- | --- | --- | --- | --- | --- |
|  | No. of sample | No. of seropositive | No. of seronegative | No. of farms | Seroprevalence (%) | No. of sample | No. of seropositive | No. of seronegative | No. of farms | Seroprevalence (%) |
| Early | 104 | 47 | 57 | 14 | 45.2 | 22 | 9 | 13 | 5 | 40.9 |
| Mid | 31 | 17 | 14 | 4 | 54.8 | 20 | 13 | 7 | 4 | 65.0 |
| Late | 139 | 96 | 43 | 16 | 69.1 | 40 | 23 | 17 | 8 | 57.5 |
| **Summary** | **274** | **160** | **114** | **34** | **58.3** | **82** | **45** | **37** | **17** | **54.8** |
